# Analyzing the Interactions between Environmental Parameters and Cardiovascular Diseases Using Random Forest and SHAP Algorithms

**DOI:** 10.31083/j.rcm2411330

**Published:** 2023-11-24

**Authors:** Gianfranco Castronuovo, Gianfranco Favia, Vito Telesca, Andrea Vammacigno

**Affiliations:** ^1^School of Engineering, University of Basilicata, 85100 Potenza, Italy; ^2^School of Medicine: Interdisciplinary of Medicine, University of Bari, 70124 Bari, Italy

**Keywords:** hospital admissions, cardiovascular diseases, climate, time series decomposition, random forest, XAI - eXplainable Artificial Intelligence techniques, feature importance

## Abstract

**Background:**

Cardiovascular diseases (CVD) remain the predominant global cause of mortality, with both low and high temperatures increasing CVD-related mortalities. Climate change impacts human health directly through temperature fluctuations and indirectly via factors like disease vectors. Elevated and reduced temperatures have been linked to increases in CVD-related hospitalizations and mortality, with various studies worldwide confirming the significant health implications of temperature variations and air pollution on cardiovascular outcomes.

**Methods:**

A database of daily Emergency Room admissions at the Giovanni XIII Polyclinic in Bari (Southern Italy) was developed, spanning from 2013 to 2019, including weather and air quality data. A Random Forest (RF) supervised machine learning model was used to simulate the trend of hospital admissions for CVD. The Seasonal and Trend decomposition using Loess (STL) decomposition model separated the trend component, while cross-validation techniques were employed to prevent overfitting. Model performance was assessed using specific metrics and error analysis. Additionally, the SHapley Additive exPlanations (SHAP) method, a feature importance technique within the eXplainable Artificial Intelligence (XAI) framework, was used to identify the feature importance.

**Results:**

An $R^{2}$ of 0.97 and a Mean Absolute Error of 0.36 admissions were achieved by the model. Atmospheric pressure, minimum temperature, and carbon monoxide were found to collectively contribute about 74% to the model’s predictive power, with atmospheric pressure being the dominant factor at 37%.

**Conclusions:**

This research underscores the significant influence of weather-climate variables on cardiovascular diseases. The identified key climate factors provide a practical framework for policymakers and healthcare professionals to mitigate the adverse effects of climate change on CVD and devise preventive strategies.

## 1. Introduction

Cardiovascular diseases (CVD) are the leading cause of global mortality, surpassing all other pathologies [1]. The Intergovernmental Panel on Climate Change (IPCC) has indicated that climate change is likely to impact human health directly through temperature fluctuations and indirectly through changes in disease vectors, such as mosquitoes, and other factors [2]. Notably, the frequency of both cold waves and heat waves has been observed to increase due to climate change [3, 4, 5]. A comprehensive review of the existing scientific literature has revealed that temperature increases will most likely lead to increases in morbidity and mortality related to weather conditions, with a significant portion of deaths related to cardiovascular events [6, 7, 8]. Many worldwide studies have confirmed extreme temperatures raise mortality risk from CVD [9, 10, 11, 12, 13], with heat waves accounting for cardiovascular-based mortality rates between 13–90% [14].

In the United States, approximately 5600 heat-related deaths occurred each year from 1997 to 2006 in 297 counties [15]. Similarly, studies conducted in 9 US cities found a 1.8% rise in mortality associated with increases in apparent temperature [16]. Additionally, in North America, for every 4.7 °C increase in daily average temperature, there is a corresponding 2.6% increase in cardiovascular mortality [17]. Furthermore, in regions where the hottest months exceeds 30 °C, it has been reported that every degree increase is associated with a 3% increase in mortality [18].

The risk of mortality from CVDs increases during both hot and cold days [19]. Similar associations between temperature and mortality have been observed in China, where there is an increased risk of mortality both at low and high temperatures [20]. For example, an analysis of the effects of ambient temperatures on mortality and morbidity in the elderly (>65 years old) showed that a 1 °C temperature increase led to a 3.44% rise in cardiovascular mortality, while a 1 °C temperature decrease resulted in a 1.66% increase in cardiovascular mortality [21].

Overall, analyses of daily mortality rates have shown that both low and high temperatures are associated with an increase in mortality from CVDs [22]. Chronic exposure to cold or heat can impair cardiovascular function leading to a higher susceptibility to heart attack, malignant cardiac arrhythmias, thromboembolic diseases, and heat-induced sepsis such as shock [23]. Changes in ambient temperature contribute to cardiovascular mortality by elevating blood pressure, blood viscosity, and heart rate [23]. Most deaths from heat waves occur in individuals with preexisting chronic CVD [23]. Seasonal variations in CVDs pose a significant health concern, with increases in hospitalization and fatal events observed during certain periods of the year, particularly in winter [24]. Counterintuitively, this phenomenon may be more problematic in populations with milder climates that are less adapted to extreme weather changes through the year [24]. Seasonality significantly influences the incidence of almost all subtypes of CVD [24]. It has been consistently demonstrated that winter is associated with a substantial increase in cardiovascular anomalies and cardiac deaths, particularly in the northern hemisphere where temperatures are particularly cold [12, 25]. Additionally, daily rates of cardiovascular events rise as mean air temperature decreases, with a 10 °C decrease associated with a 19% increase in daily rates of cardiovascular events for individuals over the age of 65 [26]. Notably, there is a strong positive correlation between maximum temperature and mortality (r = 0.83, *p* < 0.01), along with a weak but significant negative association between minimum temperatures and mortality [1].

Climate change not only impacts temperatures but also has adverse effects on other environmental conditions, particularly air pollution [27]. It was estimated that air pollution was responsible for at least 9 million global deaths in 2019 according to the Global Burden of Disease study [28]. Alarmingly, World Health Organization (WHO) data indicates that nearly the entire global population breathes air that exceeds WHO guideline limits and contains high levels of pollutants [29]. In urban areas, the impact is even more significant, as climate change affects outdoor air pollution due to its influence on the generation and dispersion of pollutants, closely linked to local patterns of temperature, wind, and precipitation [30]. These environmental changes are already causing quantifiable and avoidable acute CVD events and should be integrated into our efforts to prevent and treat CVDs [31].

In recent research at Policlinico Giovanni XXIII in Bari [32], artificial intelligence (AI) techniques demonstrated their potential by using climatic data to simulate CVD. Specifically, using feature importance techniques derived from the Random Forest algorithm, meteorological variables like mean temperature, maximum temperature, apparent temperature, and relative humidity were identified as key predictors of CVD hospitalizations. These findings highlight the potential for AI to model relationships between climatic conditions and CVD occurrences.

The primary objective of this study was to investigate the impact of climate variables on CVD and to use the variables to develop a preventive intervention framework to safeguard human health. To tackle this challenge, we propose a prescribed scheme that leverages AI methods to simulate and comprehend the relationship between climatic conditions and occurrences of CVDs. The proposed approach recognizes the multifaceted nature of this issue and takes into consideration the most pertinent meteorological variables, including mean temperature, maximum temperature, apparent temperature, and relative humidity. By employing feature importance techniques based on the Random Forest algorithm, our study identifies the crucial climatic factors that contribute to hospitalizations due to CVDs. This comprehensive understanding of the interplay between weather-climate variables and cardiovascular pathologies will enable us to identify vulnerable populations and formulate targeted preventive intervention strategies. In essence, this new proposed approach offers a practical framework for policymakers and healthcare professionals to mitigate the detrimental effects of climate change on cardiovascular health. Through the integration of AI methods and climate data, this study contributes to an enhanced comprehension of the underlying mechanisms and facilitates the development of effective preventive measures.

## 2. Materials and Methods

### 2.1 Study Area and Data Collection

This study utilized clinical records from the daily emergency department admissions of the Polyclinic hospital in the city of Bari during the reference period from 2013–2021. The database of daily hospitalizations recorded under the “main problem” field included patient cases related to the presented pathology upon their arrival at the emergency department. This database was compiled based on 33 codes, representing the specific pathologies as listed in Table 1.

**Table 1.** **Main symptoms entering the Emergency Room and identification code**.

Code	Main Problems/Symptomatology	Code	Main Problems/Symptomatology
1	Coma	18	Otorhino laryngeal symptoms or disorders
2	Acute neurological syndrome	19	Obstetric-gynecological symptoms or disorders
3	Other nervous system symptoms	20	Dermatological symptoms or disorders
4	Abdominal pain	21	Odontostomatological symptoms or disorders
5	Chest pain	22	Urological symptoms or disorders
6	Dyspnea	23	Other symptoms or disorders
7	Pre cordial pain	24	Legal-medical investigations
8	Shock	25	Social problem
9	Non-traumatic hemorrahage	26	Fall from high
10	Trauma	27	Scalding
11	Intoxication	28	Psychiatric
12	Fever	29	Pneumology-respiratory pathology
13	Allergic reaction	30	Violence from other
14	Changes in rhythm	31	Self-harm
15	Hypertension	98	Dehydration
16	Psychomotor agitation	99	Animal bite
17	Eye symptoms or disorders		
			

**Fig. 1.**
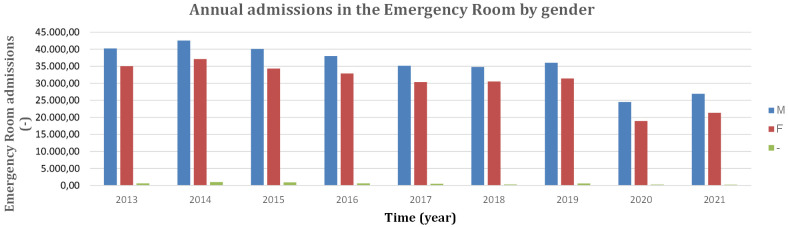
**Annual admissions in the Emergency Room by gender from 2013 to 2021**. M, male; F, female; -, gender not declared.

The first phase of data processing and reorganization was carried out by dividing the incoming information by the various examined years. The statistics presented below refer to the years 2013, 2014, 2015, 2016, 2017, 2018, 2019, 2020, and 2021. In particular, for the year 2013, there were 75,927 emergency department admissions, including 40,265 male patients, 35,032 female patients, and 630 cases of missing data regarding sex. For the year 2014, there were 80,690 total admissions, including 42,554 male patients, 37,127 female patients, and 1009 cases of missing data regarding sex. For 2015, there were 75,334 total admissions, including 40,091 male patients, 34,327 female patients, and 916 cases of missing data regarding sex. For the year 2016, there were 71,550 total admissions, including 38,007 male patients, 32,914 female patients, and 629 cases of missing data regarding sex. For 2017, there were 65,984 total admissions, including 35,130 male patients, 30,343 female patients, and 511 cases of missing data regarding sex. For 2018, there were 65,641 total emergency department admissions, including 34,798 male patients, 30,544 female patients, and 299 cases of missing data regarding sex. For 2019, there were 68,052 total admissions, including 36,034 male patients, 31,442 female patients, and 576 cases of missing data regarding sex. For 2020, there were 43,729 total admissions, including 24,517 male patients, 18,960 female patients, and 252 cases of missing data regarding sex. In 2021, the total number of Emergency Room (ER) admissions amounted to 48,489, including 26,904 male patients, 21,355 female patients, and 230 cases of missing data regarding sex (Table 2, Fig. 1).

**Table 2.** **Admissions in the Emergency Room (ER) by gender from 2013 to 2021**.

	2013	2014	2015	2016	2017	2018	2019	2020	2021
Total admissions in the ER	75,927	80,690	75,334	71,550	65,984	65,641	68,052	43,729	48,489
Male	40,265	42,554	40,091	38,007	35,130	34,798	36,034	24,517	26,904
Female	35,032	37,127	34,327	32,914	30,343	30,544	31,442	18,960	21,355
Undeclared gender	630	1009	916	629	511	299	576	252	230

For the purpose of this study, only data and statistics both related to cardiovascular pathologies and are strongly correlated with meteorological factors (Table 3).

**Table 3.** **Classification of the specific problems entering in ER into main diseases**.

Code	Specific Problem	Classification
5	Chest pain	Cardiovascular diseases
7	Precordial pain	
14	Changes in rhythm	
15	Hypertension	

ER, Emergency Room.

**Fig. 2.**
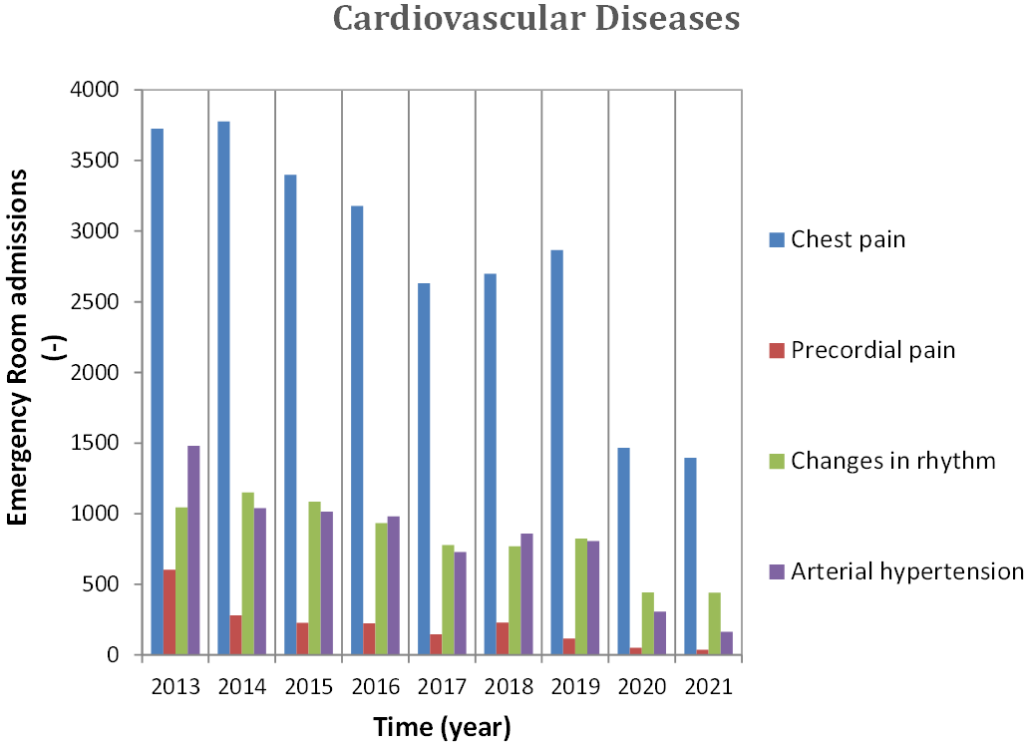
**Annual admission in ER for CVD, 2013–2021**. CVD, cardiovascular diseases; ER, Emergency Room.

**Fig. 3.**
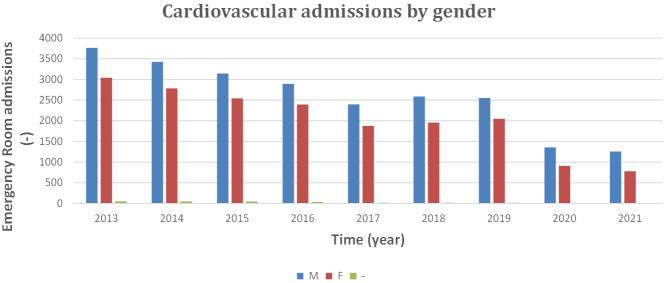
**Admissions in ER for CVD classified by gender, 2013 to 2021**. CVD, cardiovascular diseases; ER, Emergency Room; M, male; F, female.

**Fig. 4.**
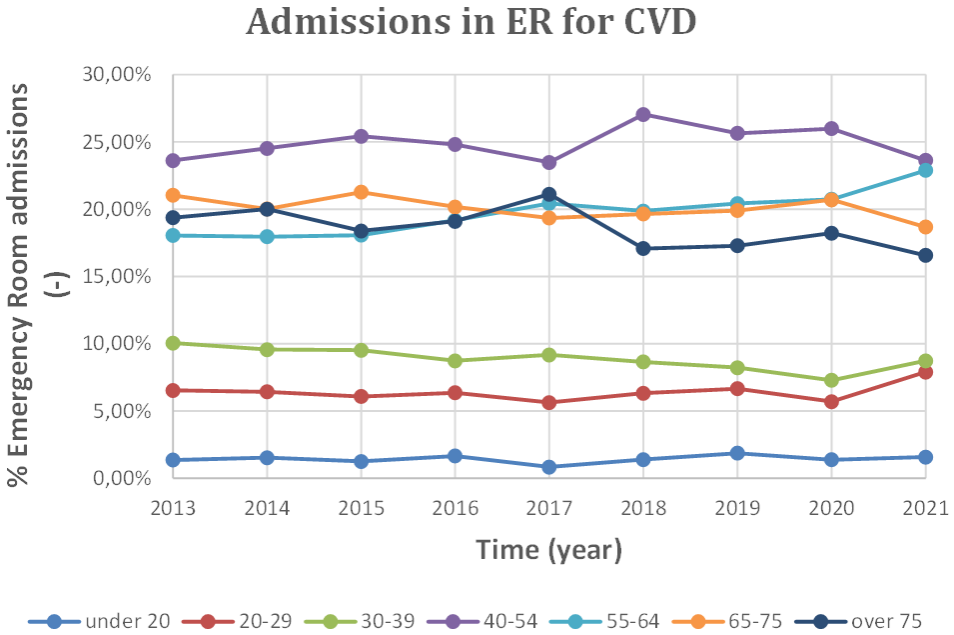
**Admissions in ER for CVD by age, 2013 to 2021**. ER, Emergency Room; CVD, cardiovascular diseases.

Analysis of the effects of hot and cold ambient temperatures on mortality and morbidity in the elderly (>65 years old) showed that a 1 °C increase in temperature increased cardiovascular mortality (3.44%), while a 1 °C decrease in temperature increased cardiovascular mortality (1.66%) [23]. Therefore, out of the 33 types of pathologies described in Table 1, only those related to CVDs were selected according to the grouping scheme reported in Table 3 and analyzed for the various years examined, as shown in Fig. 2. The effects of climate variations may have different effects on each individual. A first step in identifying whether there are substantial differences among different types of individuals is gender analysis. Table 4 and Fig. 3 show the annual Emergency Room admissions for cardiovascular pathologies by gender from 2013 to 2021. A further and important distinction is made according to the age of the patient. The distribution of cardiovascular admissions in the emergency department is shown in Tables 5a,5b and in Fig. 4 as a percentage of total admissions.

**Table 4.** **Admissions in ER for CVD classified by gender, 2013 to 2021**.

		2013	2014	2015	2016	2017	2018	2019	2020	2021
Cardiovascular diseases	Admissions in the ER	6854	6252	5728	5319	4284	4558	4615	2268	2040
	Male	3762	6422	3143	2893	2396	2586	2548	1353	1256
	Female	3040	2781	2540	2393	1873	1955	2050	908	778
	Undeclared gender	52	49	45	33	15	17	17	7	6

ER, Emergency Room; CVD, cardiovascular diseases.

**Table 5a.** **Admissions in ER for CVD classified by age range (Absolute numbers)**.

	Admissions in Emergency Room for cardiovascular diseases								
Year	under 20	20–29	30–39	40–54	55–64	65–75	over 75	tot	tot admission
2013	92	447	688	1617	1236	1440	1326	6846	75,927
2014	95	401	597	1532	1122	1251	1250	6248	80,690
2015	71	348	545	1456	1035	1218	1053	5726	75,334
2016	88	338	464	1320	1020	1073	1016	5319	71,550
2017	35	238	388	994	865	819	894	4233	65,985
2018	63	288	394	1232	905	895	778	4555	65,641
2019	86	307	379	1183	942	918	797	4612	68,052
2020	31	129	165	589	470	469	413	2266	43,729
2021	32	161	178	482	467	381	338	2040	48,489

ER, Emergency Room; CVD, cardiovascular diseases.

**Table 5b.** **Admissions in ER for CVD classified by age range (Percentages)**.

	Emergency Room admissions (%) for cardiovascular diseases							
Year	under 20	20–29	30–39	40–54	55–64	65–75	over 75	tot
2013	1.34	6.53	10.05	23.62	18.05	21.03	19.37	9.02
2014	1.52	6.42	9.56	24.52	17.96	20.02	20.01	7.74
2015	1.24	6.08	9.52	25.43	18.08	21.27	18.39	7.60
2016	1.65	6.35	8.72	24.82	19.18	20.17	19.10	7.43
2017	0.83	5.62	9.17	23.48	20.43	19.35	21.12	6.42
2018	1.38	6.32	8.65	27.05	19.87	19.65	17.08	6.94
2019	1.86	6.66	8.22	25.65	20.42	19.90	17.28	6.78
2020	1.37	5.69	7.28	25.99	20.74	20.70	18.23	5.18
2021	1.57	7.89	8.73	23.63	22.89	18.68	16.57	4.21

ER, Emergency Room; CVD, cardiovascular diseases.

As shown in Fig. 4, for cardiovascular diseases, the age group between 40 and 54 years has the highest number of hospitalizations.

### 2.2 Meteo-Climatic Parameters

The meteo-climatic parameters considered in this study include: daily mean minimum temperature (Tmin), daily mean temperature (Tmean), daily mean maximum temperature (Tmax), daily mean dew point temperature (Tdewp), daily mean apparent temperature (Tapp), daily mean atmospheric pressure (Patm), and daily mean relative humidity (RH). Air quality parameters considered include CO (carbon monoxide), $O_{3}$ (ozone), PM10 (particulate matter), ${\mathrm{SO}}_{2}$ (sulfur dioxide), and ${\mathrm{NO}}_{2}$ (nitrogen dioxide) (Table 6). The meteorological data for the city of Bari for the reference period 2013–2021 were obtained from the Arpa Puglia website and the Meteonetwork measurement network. Arpa Puglia manages two networks for monitoring activities:

∙ A dedicated network: 5 automatic stations located at its provincial offices (Bari, Brindisi, Foggia, Lecce, and Taranto).

∙ A meteorological network supporting the air quality monitoring network currently consisting of 19 stations.

**Table 6.** **Descriptive data statistics**.

	Tmin	Tmean	Tmax	Tdewp	Tapp	P_atm	RH	CO	$O_{3}$	PM10	${\mathrm{SO}}_{2}$	${\mathrm{NO}}_{2}$	CVD
min	–0.17	–0.11	–0.04	–6.38	2.96	976.60	25.49	0.10	13.00	1.00	0.00	5.00	0
avg	17.08	17.78	18.51	11.92	23.32	1010.93	70.22	0.77	83.51	22.54	17.41	52.96	12.9
max	32.86	33.59	41.60	26.00	52.78	1039.35	99.0	3.00	154.0	117.00	104.0	157.0	37
std	6.40	6.40	6.70	5.52	8.74	8.25	10.89	0.41	21.09	11.30	21.41	25.65	6.3
75th	22.50	23.34	23.92	16.49	30.25	1016.38	78.0	1.00	99.00	27.00	26.90	69.00	17
50th	16.65	17.20	17.90	12.00	22.19	1011.55	71.0	0.70	83.00	21.00	6.90	50.00	13
25th	11.80	12.40	13.12	7.82	15.80	1005.30	62.95	0.50	68.00	15.00	3.10	33.00	8

Legend: Tmin, minimum temperature; Tmean, mean temperature; Tmax, maximum temperature; Tdewp, dew point; Tapp, apparent temperature; P_atm, atmospheric pressure; RH, relative humidity; CO, carbon monoxide; $O_{3}$, ozone; PM10, Particulate Matter smaller than about 10 micrometers; ${\mathrm{SO}}_{2}$, sulfur dioxide; ${\mathrm{NO}}_{2}$, nitrogen dioxide; CVD, cardiovascular diseases; avg, average; std, standard deviation; min, minimum; max, maximum; 25% 50% and 75% 25th 50th and 75th percentile respectively, min–max = range.

The dedicated Bari station also monitors ultraviolet radiation [33]. Air quality data for the city of Bari from 2013 to 2021 was obtained from the Arpa Puglia website, through the Bari-Caldarola, Bari-CUS, Bari-Kennedy, Bari-Carbonara monitoring stations, and the mobile laboratory. Meteorological data are recorded with a half-hourly frequency for the Arpa Puglia weather stations and with a daily frequency of five minutes and one hour for the Meteonetwork measurement network. The hourly frequency concerns only the years 2020 and 2021. Air quality data were recorded on a daily basis. The apparent temperature and ${\mathrm{SO}}_{2}$ (sulfur dioxide) were excluded from the meteorological and air quality parameters, respectively, due to a lack of representative data, which is insufficient for a complete and accurate elaboration of the same.

### 2.3 Methodology

**Fig. 5.**
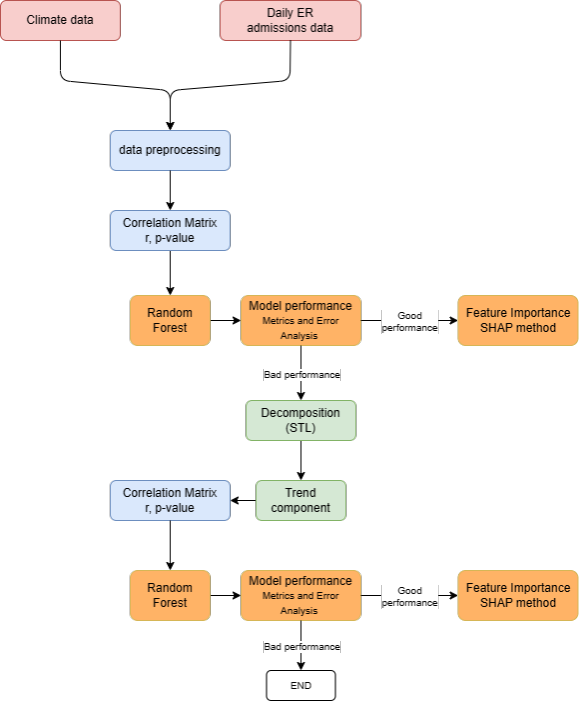
**Methodology**. ER, Emergency Room; r, Pearson correlation coefficient; SHAP, SHapley Additive exPlanations; STL, Seasonal and Trend decomposition using Loess.

The primary objective of the study was to determine which meteorological and air quality variables have the greatest impact on emergency department admissions for CVDs. The methodology adopted from Telesca *et al*. [32] (Fig. 5). The first step was to find correlations between meteorological variables and emergency department admissions for CVDs through correlation analysis (see Section 4.2), calculating the Pearson coefficient “r” and *p*-value. Subsequently, the Random Forest model was applied along with its corresponding metrics to evaluate the model’s performance. If the analysis produced acceptable results (Mean Absolute Error [MAE], coefficient of determination $R^{2}$, and error analysis) further analysis was performed to determine the most significant meteorological variables (see Section 4.4). If the results were not acceptable, a data decomposition model (Seasonal and Trend decomposition using Loess (STL) - see Section 4.3) is applied to extract the trend component from the data series. Then, the correlation analysis is repeated. This iterative process eventually leads to the most significant variables for the final correlation.

### 2.4 Theoretical Aspects of Random Forest Techniques

Random Forest, a powerful ensemble learning method, is built upon decision trees, which are hierarchical structures used for making sequential decisions in assigning data points to classes or predicting continuous values in regression tasks. By combining the predictions of multiple models, Random Forest achieves accurate regression predictions. Each model in the ensemble represents a decision tree, and together, they leverage collective knowledge to enhance the overall predictive capability. To construct decision trees, the algorithm employs bootstrap aggregating, also known as bagging. This technique involves creating multiple bootstrap samples from the original training data. Each sample is obtained by randomly selecting data points with replacement, forming subsets used to train individual decision trees. In addition to bagging, Random Forest introduces randomness in feature selection during decision tree construction. Instead of considering all features at each node, a random subset of features is chosen for splitting. This approach ensures that each decision tree in the ensemble learns and makes predictions based on different aspects of the data, leading to a diverse and robust ensemble. When generating predictions with a Random Forest regression model, the ensemble combines the individual decision tree predictions. A commonly employed approach is to compute the average of the predicted values across all trees. This averaging technique helps mitigate the impact of outliers and noise, yielding more reliable predictions. Random Forest provides a convenient mechanism for estimating the model’s performance without requiring a separate validation set. This mechanism relies on out-of-bag (OOB) samples, which are data points that were left out in each bootstrap sample. By comparing the predictions of these OOB samples with their actual target values, one can evaluate the model’s accuracy. Moreover, Random Forest offers insights into the relative importance of different features in the regression task. By analyzing the impact of feature splits across the ensemble of decision trees, the model calculates variable importance scores. These scores indicate which features contribute the most to the model’s predictive power, providing interpretability and understanding of the underlying data relationships. In brief, there are various theoretical advantages associated with using Random Forest techniques for regression models. These include ensemble learning using decision trees, bagging for robust tree construction, random feature selection to ensure diversity, and the ability to estimate model performance and assess variable importance. Collectively, these aspects contribute to the overall effectiveness, accuracy, and interpretability of Random Forest as a powerful regression modeling technique.

### 2.5 Preprocessing of Data

**Fig. 6.**
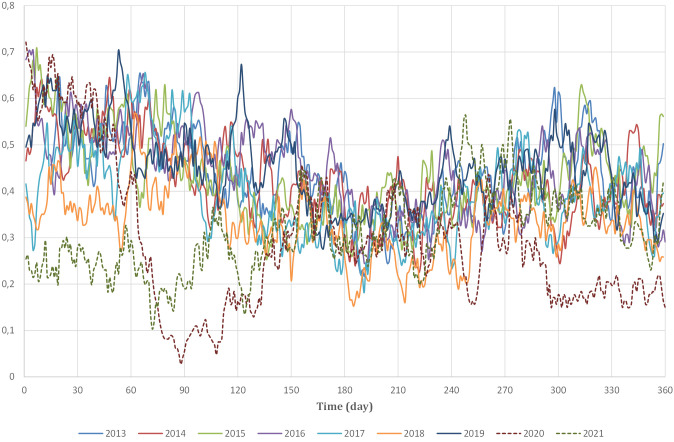
**Year-to-year 7-day moving averages in Emergency Room admissions, 2013 to 2021**.

The initial step of the process involved removing records with missing data from the database to ensure data integrity. This was followed by searching for the trends in hospital admissions between 2013 to 2021 to account for possible interference caused by anomalous variations in ER visits during the pandemic years of 2020 and 2021. The exclusion of data from 2020 and 2021 was based on the analysis showing distinct differences in ER visits during those years, as depicted in Fig. 6, which presents the 7-day moving averages normalized with the minimum/maximum (min/max) method to ensure comparability across different years. Thus, only data from the years 2013 to 2019 were considered for further analysis.

## 3. Data Modeling

### 3.1 Correlation Analysis

Correlation analysis is a bivariate statistical technique that measures the strength of a linear relationship between two variables and calculates this relationship. In order to express quantitatively the intensity of the link between two variables, it is necessary to calculate a correlation coefficient [34]. There are various types of correlation coefficients, but all have some common features such as the value that oscillates between –1 and +1, representing a perfect relationship between variables. While a value of 0 indicates the absence of a relationship [35]. To conduct this analysis, a correlation matrix was used. The correlation matrix is a table in which each cell shows the correlation between two variables. The matrix calculates the Pearson correlation coefficient “r”, one of the most widely used correlation coefficients, for each pair of characteristics.

**Fig. 7.**
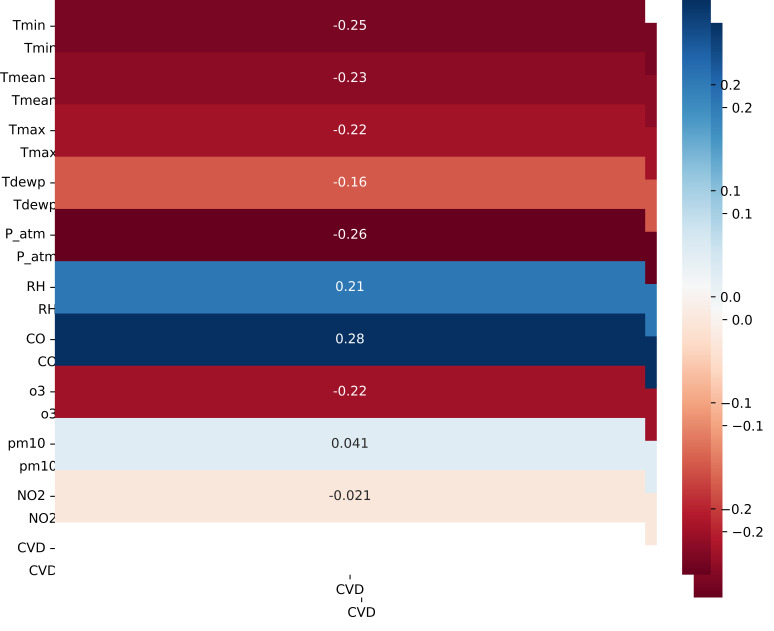
**Correlation between features and CVD**. Tmin, minimum temperature; Tmean, mean temperature; Tmax, maximum temperature; Tdewp, dew point; P_atm, atmospheric pressure; RH, relative humidity; CO, carbon monoxide; $O_{3}$, ozone; PM10, Particulate Matter smaller than about 10 micrometers; ${\mathrm{NO}}_{2}$, nitrogen dioxide; CVD, cardiovascular diseases.

Correlation analysis was conducted utilizing the *p*-value as a tool for significance testing of the null hypothesis. The *p*-value represents the probability of obtaining a specific set of observed values assuming the null hypothesis is true, indicating the correctness of our assertion with minimal error. A significantly small *p*-value indicates that an extreme observed result would be highly improbable under the null hypothesis. This statistical measure establishes the reliability of the correlation values obtained. Acceptable hypotheses for the input variable set were defined as having *p*-values less than 0.01 and r-values greater than or equal to 0.45 [36]. In the context considered, none of the input variables of the model meet the above conditions, as evidenced by the correlation matrix (Fig. 7). Therefore, the decomposition model will be used.

### 3.2 Decomposition Model

Many forecasting methods are based on the idea that if there is a systematic pattern, it can be identified and separated from any random fluctuations by smoothing methods of the historical series data. The smoothing effect is the removal of random disturbances, once the pattern is known, in order to project it into the future for forecasting purposes. Decomposition models are mainly divided into additive time series models and multiplicative time series models. The additive model assumes that the effects of each component are independent of each other and that each component is expressed in absolute terms. An additive model is appropriate when the amplitude of the seasonal oscillation does not vary with the level of the series. The error can take positive or negative values, while the neutral value is expressed with the value 0, meaning it does not affect the series. The multiplicative model assumes that the effects of each component on the evolution of the phenomenon are interrelated based on the absolute magnitude of the trend component and that the other components are expressed proportionally. A multiplicative model is suitable when the seasonal fluctuation varies, increases, or decreases proportionally with the variation of the series level. The error can only take non-negative values and has a neutral value of 1. STL is a statistical method that allows the decomposition of time series data into three components: trend, seasonality, and residual. The trend component reflects the long-term variation of the series. A trend exists when there is a persistent increasing or decreasing direction in the data. The seasonal component reflects seasonality, i.e., the variation of data that occurs at specific regular intervals of one year or less. The residual component describes casual or irregular influences. It represents the residuals of the series after the other components have been removed [37].

Let $Y_{v}$, $T_{v}$, $S_{v}$e $R_{v}$, for v = 1 to n, represent the data, trend component, seasonal component, and residual component, respectively (Eqn. 1).

(1) $$\begin{array}{c} Y_{v}=T_{v}+S_{v}+R_{v} \end{array}$$

**Fig. 8.**
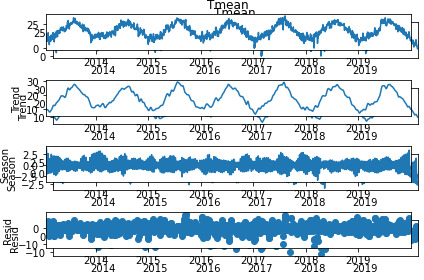
**Example of a decomposition of mean daily temperature from 2013 to 2021 into the three components: trend, seasonal, and residual**.

The STL consists of a sequence of smoothing operations, each of which, with one exception, employs the same smoother: locally weighted regression, or locally estimated scatterplot smoothing (Loess). Local regression or Loess involves determining, for each point in the initial data set, the coefficients of a low-degree polynomial to perform regression on a subset of data and calculate the value of this polynomial for the given point. The coefficients of the polynomial are computed using weighted least squares, which gives more weight to points near the point whose response is being estimated and less weight to more distant points. This method is used to fit the entire curve and accurately decompose the time series [38]. Fig. 8 shows an example of the decomposition, using the STL model, of the daily average temperature from 2013 to 2021 examined in this thesis. Four graphs are shown:

∙ The first is the original series;

∙ The second is the trend;

∙ The third is the seasonality;

∙ The fourth is the residual.

**Fig. 9.**
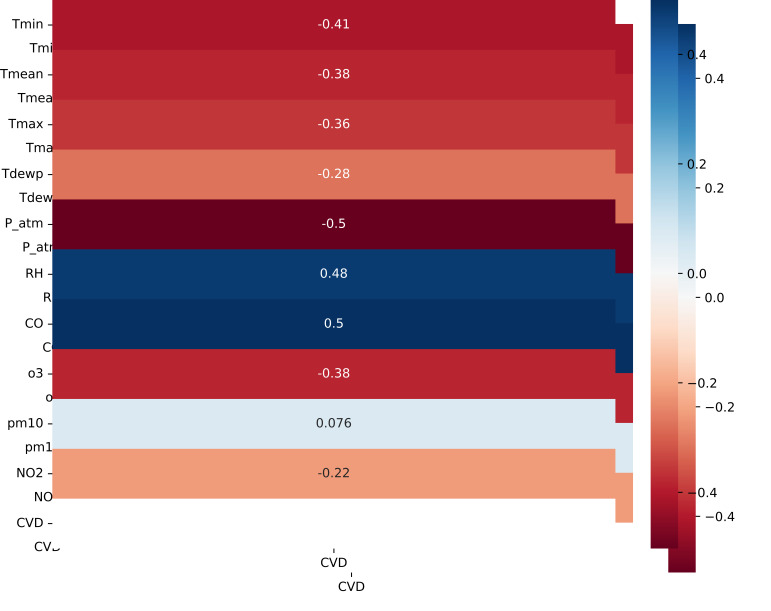
**Correlation between features and CVD after decomposition model application**. Tmin, minimum temperature; Tmean, mean temperature; Tmax, maximum temperature; Tdewp, dew point; P_atm, atmospheric pressure; RH, relative humidity; CO, carbon monoxide; $O_{3}$, ozone; PM10, Particulate Matter smaller than about 10 micrometers; ${\mathrm{NO}}_{2}$, nitrogen dioxide; CVD, cardiovascular diseases.

As for our study, the STL decomposition model was applied to the original data after verifying the stationarity of the series using the Dickey-Fuller method [39]. The Dickey-Fuller test is a statistical method used to check whether a time series is stationary or not, i.e., if its statistical properties do not change over time. Thus, a modified series was obtained, represented by the trend values of the original data. Then, by recalculating the correlation matrix, significantly higher Pearson coefficient values were obtained compared to the original data series (Fig. 9). These results allow the application of machine learning models to produce an effective simulation model of the trend of hospital admissions over time.

### 3.3 Application of the Machine Learning Model

Random Forest is the AI model used to simulate the trend of emergency department visits for the two considered pathologies, and was also estimated the most influential meteorological variables. The Random Forest is a supervised machine learning algorithm, a special classifier consisting of a set of simple classifiers, called decision trees, represented as independent and identically distributed random vectors. Decision trees are common supervised learning algorithms. Decision trees start with a basic question to determine an answer, and these questions make up the decision nodes of the tree that serve to divide the data. Each question helps to make a final decision, which is indicated by the leaf node. When multiple decision trees form an ensemble in the Random Forest algorithm, they predict more accurate results, particularly when the individual trees are not correlated with each other. An ensemble refers to a set of decision trees, and their predictions are aggregated to identify the most popular result [40]. The Random Forest combines the results of all these decision trees to obtain a single result. Each decision tree is composed of a data sample drawn from a training set with replacement, which means that individual data points can be chosen more than once. From this training sample, one-third is set aside as test data, also known as an OOB sample. Another instance of randomness is injected through feature begging or the random subspace method, adding greater diversity to the data set and reducing the correlation between decision trees. Depending on the type of problem, the determination of the forecast varies. For a regression task, the individual decision trees will be averaged, while for a classification task, the majority vote will give the prediction class. Finally, the test sample is used for cross-validation, finalizing the prediction. Cross-validation is a statistical technique that allows the use of both training and test data alternately. There are several cross-validation methods, and the K-fold was used in this paper. K-fold divides the data into k different subsets and k-1 are used for the training phase and the last, remaining subset, for the test phase. The error is then calculated on the observations of the subsets kept out. This procedure is repeated k times by choosing a different subset and obtaining k estimates of the test error. The final estimate will be an average of these values [41].

### 3.4 Model Performance Analysis

Table 7 reports the values of the MAE and $R^{2}$ metrics for the sample extracted during training and testing phases, as well as the same metrics in the average value of the cross-validation subsets.

**Table 7.** **Values of the MAE and $R^{2}$ metrics (sample extracted and cross validation)**.

	$R^{2}$ train	$R^{2}$ test	MAE train	MAE test
CVD with STL	0.996	0.969	0.13	0.36
Cross validation (mean values)	0.996	0.973	0.12	0.33

Legend: MAE, mean absolute error; STL, Seasonal and Trend decomposition using Loess; CVD, cardiovascular diseases.

**Fig. 10.**
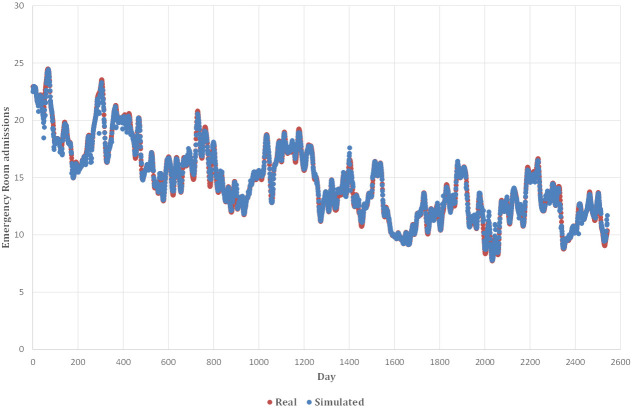
**Real and simulated data from the model for CVD**. CVD, cardiovascular disease.

The values obtained through cross-validation are analogous to those determined for the random sample, highlighting the model’s ability to avoid overfitting phenomena, demonstrating the accuracy of the model. Subsequently, in Fig. 10, it is shown in the left graph how the trend of the data concerning cardiovascular pathologies predicted by the Random Forest model, described in blue, has an identical trend to the actual data.

**Fig. 11.**
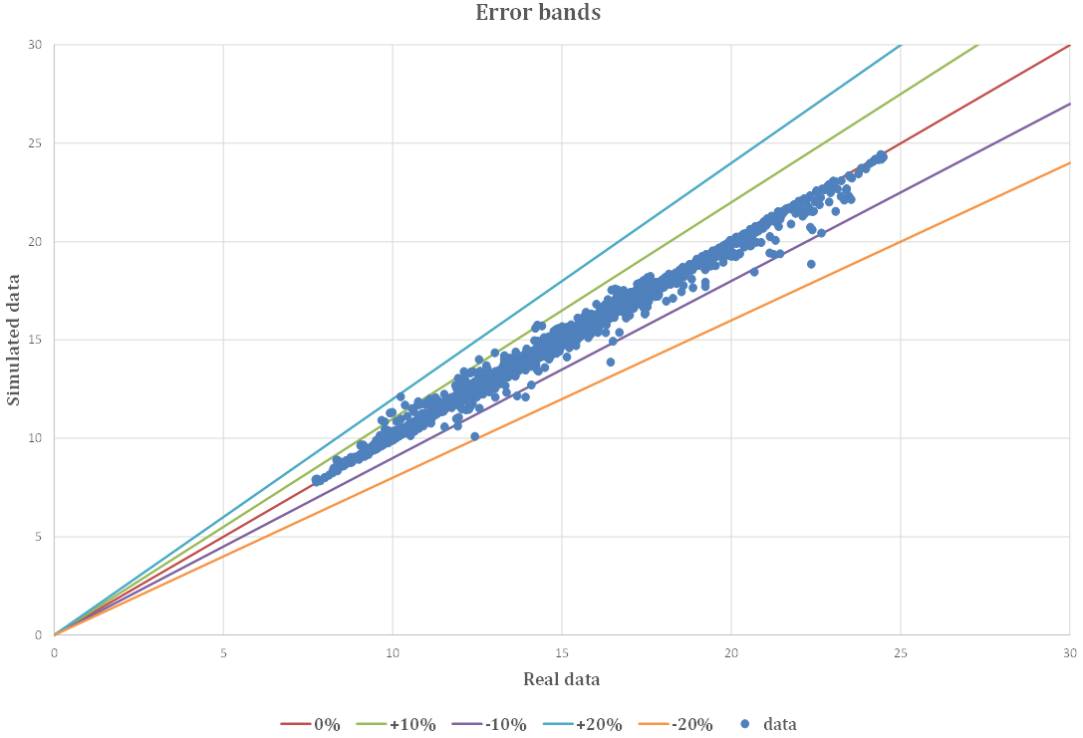
**Real and simulated data with error bands from the model**.

The accuracy of the model is highlighted in Fig. 10. The blue points represent both the real and simulated data, and for the most part, they fall within the error bands (Fig. 11). Most data points fall within ± 10% of the error bands and only a few values fall within ± 20% of the error bands. In particular, 2443 out of 2542 data points (total number of data points, 96.10% of the total number of ER admissions), fall within the ± 5% error band (Table 8).

**Table 8.** **Data number between error bands**.

Error X (%)	Data numbers and relative %
X <–20	1 (0.04)
–20 < X < –15	3 (0.12)
–15 < X < –10	7 (0.28)
–10 < X < –5	34 (1.34)
–5 < X < 0	1189 (46.77)
0 < X < +5	1254 (49.33)
+5 < X < +10	48 (1.89)
+10 < X < +15	5 (0.20)
+15 < X < +20	1 (0.04)
X >+20	0

### 3.5 Feature Importance

**Fig. 12.**
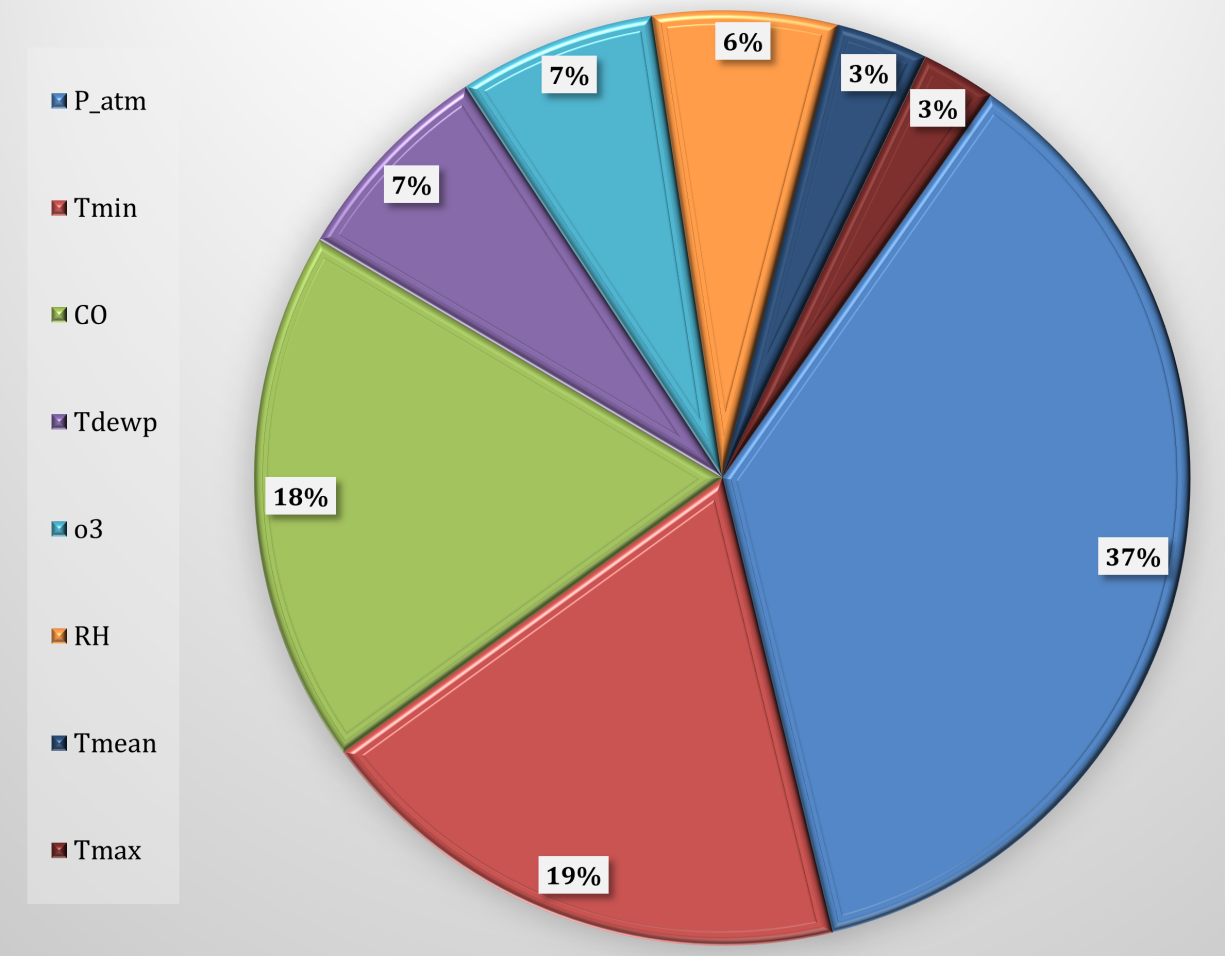
**Feature importance selected for CVD**. CVD, cardiovascular diseases; P_atm, atmospheric pressure; Tmin, minimum temperature; CO, carbon monoxide; Tdewp, dew point; RH, relative humidity; $O_{3}$, ozone; Tmean, mean temperature; Tmax, maximum temperature.

Since the Machine Learning simulation model has been shown to provide accurate results, we can proceed with the use of Feature Importance techniques through the SHapley Additive exPlanations (SHAP) method to identify the most influential characteristics in determining the trend of daily hospital admissions for CVDs, according to the block diagram of the methodology (Fig. 5). Feature Importance shows how the most characteristic variables for CVDs, Fig. 12, are atmospheric pressure with a percentage of 37%, minimum temperature with 19%, and carbon monoxide (CO) with a percentage of 18%. These three variables represent about 74% of the model’s predictability, with atmospheric pressure being predominant.

## 4. Results

### SHAP Feature Importance

**Fig. 13.**
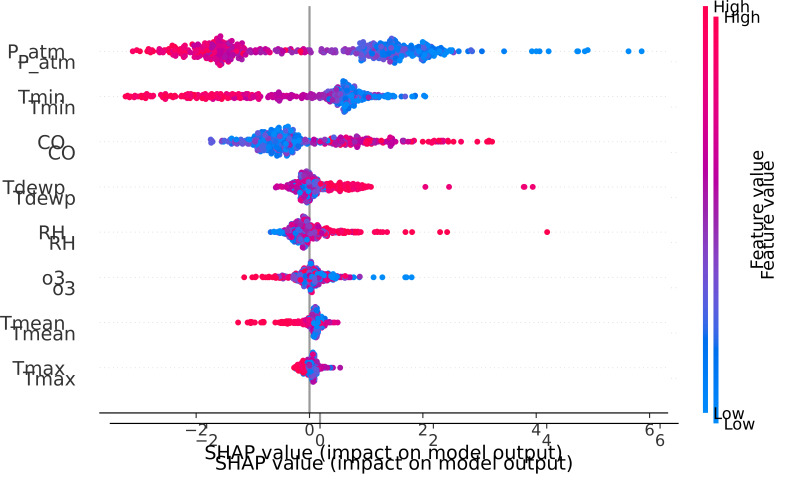
**Feature importance (SHAP model)**. Legend: SHAP, SHapley Additive exPlanations; P_atm, atmospheric pressure; Tmin, minimum temperature; CO, carbon monoxide; Tdewp, dew point; RH, relative humidity; $O_{3}$, ozone; Tmean, mean temperature; Tmax, maximum temperature.

The SHAP model integrated and analyzed cumulative datasets derived from the previous models in this study. This data was visualized using bee swarm plot diagrams, as illustrated in Fig. 13. While the features are ranked by their contributed weight to the prediction, with consideration given to the range and value (i.e., ±) in the model.

The SHAP method provides insights into both the global and local contributions of each variable, with the results presented through an importance ranking visualized through a bee swarm scatter plot. Within this representation, the horizontal axis signifies the SHAP value, while the color of the point indicates the intensity of the values (blue for low values, red for high values) of each variable.

The SHAP model identified the variables i with the most pronounced impact on hospital admissions. These variables are arranged in descending order of importance as follows: Atmospheric Pressure (P_atm), Minimum Temperature (Tmin), and Carbon Monoxide (CO) concentration. In Fig. 13, the correlation between low values of P_atm and Tmin and their correspondence to elevated SHAP values (indicating significance) is readily discernible. This graphical representation underscores their substantial role in enhancing the effectiveness of the simulation model. Conversely, the behavior of CO exhibits the opposite pattern, increasing in importance for higher values.

The remaining variables were found to exhibit less significance. These include: Dew Point (Tdewp), Relative Humidity (RH), Ozone ($O_{3}$), and Mean Temperature (Tmean). On the other hand, Tmax provides a nearly negligible contribution, regardless of its values. This comprehensive analysis allows for a nuanced understanding of the varying impacts of these variables on hospital admissions.

## 5. Discussion

The aim of this study was to explore the correlations between weather and air quality factors with Emergency Room admissions for CVDs. To accomplish this goal, the Random Forest AI model, model, was used to highlight the most important characteristics. The initial data obtained from the correlation matrices, yielded unsatisfactory results. Consequently, an STL decomposition model was used to reduce the error in predicting characteristic variables. The utilization of this model significantly improved the results when the data underwent further analysis via the Random Forest model. We determined that both real and predicted error band data predominantly fell within the range of ±10%, with the exception of a few values that slightly exceeded the ±20% margin. The latter values, analyzed through the subsequent procedure, exhibited remarkably high accuracy, thus underscoring the importance of specific variables in generating predictions. These latter values, analyzed through the subsequent procedure, exhibited remarkable accuracy, thus underscoring the importance of specific variables in generating predictions. The most significant variables for CVDs were identified as atmospheric pressure (contributing over 37%), minimum temperature (19%), and carbon monoxide (CO) (18%). These three variables collectively accounted for 74% of the model’s predictability, with atmospheric pressure playing a prominent role.

### 5.1 Role of Temperature, Atmospheric Pressure and Carbon Monoxide Concentration in Simulating CVD Admissions

The results of this study provide valuable insights into the relationship between environmental factors and cardiovascular health. Our findings suggest that atmospheric pressure, minimum temperature, and carbon monoxide are the most important factors in predicting the number of hospital admissions for CVD. These results are consistent with previous studies that have identified these factors as risk factors for CVD [6, 7, 8, 9, 10, 11, 12, 13, 14, 16, 20]. Previous studies have consistently shown that extreme temperatures increase mortality from CVD [9, 10, 11, 12, 13]. This association between temperature and CVD mortality is observed for both low and high temperatures [22]. Chronic exposure to cold or heat can impair cardiovascular function, consequently increasing the risk of heart attack, arrhythmias, thromboembolic diseases, and heat-induced sepsis [23]. Furthermore, changes in ambient temperature have been shown to contribute to cardiovascular mortality by causing increased blood pressure, blood viscosity, and heart rate [23]. Seasonal variations in CVD pose a significant health challenge [24], particularly for populations residing in milder climates, who may be less adapted or prepared for extreme climate changes. A majority of studies have reported a winter increase in cardiovascular anomalies and cardiac death in the northern hemisphere, where temperatures are exceedingly cold [12, 25]. In light of this evidence, it is evident that temperature plays a crucial role in the prevalence of CVDs and should be considered a vital parameter when developing predictive models for hospital admissions related to these conditions.

Our results not only emphasize the importance of temperature but also highlight the significance of atmospheric pressure and carbon monoxide concentration in predicting hospital admissions for CVDs. These findings are consistent with existing literature. A connection between small changes in low levels of ambient carbon monoxide concentrations and cardiovascular hospitalizations has been demonstrated [42]. Exposure to carbon monoxide at concentrations found in heavy tobacco smokers or individuals with significant occupational exposure has been shown to play a role in the pathogenesis of CVD [43]. Short-term exposure to ambient carbon monoxide has been associated with an increased risk of CVD hospitalizations [42], as well as increased rates of cardiovascular and coronary heart diseases in major Chinese cities [44]. Ambient carbon monoxide levels have also been positively correlated with hospital admissions for CVDs [45]. Moreover, carbon monoxide exposure has been shown to have detrimental effects on exercise performance in subjects with coronary artery disease, further demonstrating its impact on myocardial ischemia [46]. In addition to carbon monoxide, high atmospheric pressures have been associated with an increased number of cardiac arrest admissions [47]. This evidence suggests that both atmospheric pressure and carbon monoxide concentration are crucial factors to consider when developing predictive models for hospital admissions related to CVDs. Given the growing body of research, it is crucial to incorporate temperature, atmospheric pressure, and carbon monoxide concentration into predictive models for hospital admissions due to CVDs, as these factors play a significant role in the onset and exacerbation of cardiovascular conditions.

### 5.2 A Personalized Healthcare Service Recommendation Framework for CVD Based on Meteorological Condi-tions and Air

Recent research has provided significant contributions to understanding and addressing the negative impacts of climate change on CVD. One such study [48] proposes a healthcare service recommendation framework based on an embedded user profile model. This personalized approach could be extended to the domain of CVD, enabling targeted healthcare service suggestions based on meteorological conditions and air quality. An innovative approach, presented in [49] and [50], utilizes deep learning through convolutional neural networks or ResNet-based classification models to identify the presence of diseases, such as COVID-19, from radiological images. This methodology could also be adapted for early diagnosis of CVD, correlating specific radiological signs.

Furthermore, Yu and colleagues [51] have devised an approach that aims to acquire insights into the causality of diseases and foster a more comprehensive understanding of the interconnections between symptoms and diseases. This framework shows potential in revealing causal connections between meteorological factors, air quality, and CVDs. Collectively, these studies collectively contribute to enhancing our understanding of the complex relationships between environmental variables and cardiovascular health.

Future studies should prioritize establishing alert thresholds based on these parameters, with additional efforts dedicated to expanding the scope of the model to encompass a wider array of diseases and other geographical regions. To enhance the model’s predictive prowess, validation procedures and potential refinements will be investigated. This could involve integrating data sourced from multiple hospitals situated across different geographical areas and climates. The ultimate goal is to gain access to the comprehensive regional database of Puglia hospitals to validate the model’s generalizability. This step will serve to reinforce its reliability and broaden its applicability.

In conclusion, there is a strong relationship between daily Emergency Room admissions and the variation of some weather and air quality parameters. Future studies should be directed toward pinpointing and defining alert thresholds rooted in these parameters.

## Data Availability

The data used in this study can be requested from the corresponding author: Prof. Vito Telesca at vito.telesca@unibas.it.

## Abbreviations

AI, artificial intelligence; Arpa, Agenzia Regionale per la Protezione Ambientale; CVD, cardiovascular diseases; Dewp, dew point; ER, Emergency Room; IPCC, Intergovernmental Panel on Climate Change; Loess, locally estimated scatterplot smoothing; MAE, mean absolute error; OOB, Out-of-Bag; P_atm, atmospheric pressure; $R^{2}$, R-squared; RH, relative humidity; SHAP, SHapley Additive exPlanations; STL, Seasonal and Trend decomposition using Loess; Tapp, apparent temperature; Tmax, maximum temperature; Tmean, mean temperature; Tmin, minimum temperature; WHO, World Health Organization; XAI, eXplainable Artificial Intelligence.
